# Health Promotion in Sport, through Sport, as an Outcome of Sport, or Health-Promoting Sport—What Is the Difference?

**DOI:** 10.3390/ijerph18179045

**Published:** 2021-08-27

**Authors:** Susanna Geidne, Aurélie Van Hoye

**Affiliations:** 1 Faculty of Medicine and Health, School of Health Sciences, Örebro University, SE-701 82 Örebro, Sweden; 2 APEMAC, University of Lorraine, Villers-lès-Nancy, 54600 Nancy, France; aurelie.van-hoye@univ-lorraine.fr

What do we currently know about the relationship between health promotion and sport in research? In this editorial, we will argue that it depends on how the concepts of health promotion and sport are delineated. Because of this, the relation can be more or less inclusive than expected at first glance.

For decades, the concepts of public health, health, and health promotion have been used in relation to sport in many ways in the literature, often without clear definitions or descriptions of the relationship between the concepts. If, on one hand, health promotion is defined as “the process of enabling people to increase control over, and to improve their health” [1], what will the relationship look like? If, on the other hand, sport is defined, as in this Special Issue, in a broad sense, including both competitive and recreational sport, targeting all groups across the lifespan and socio-economic gradient, including every day and non-organized sport practice, what will the relationship look like then?

In a recent review, Geidne and colleagues [2] identified an increasing number of studies in the field, embracing wider notions of the relationship between health promotion and sport. To further this development, the broad objective of this Special Issue was to illustrate different types of associations between health promotion and sport, to support a clarification of the concepts, foster the promotion of studies focusing on health promotion beyond mere physical activity promotion, and to offer space for research on structural and organizational approaches.

Having both health promotion and sport definitions in mind, the Special Issue aims to investigate four types of associations: (i) health promotion as an outcome of sport, (ii) health promotion through sport, (iii) health promotion in sport, and (iv) health-promoting sport.

Health promotion, as an outcome of sport, is based on the concept that organized sports are promoting health because they offer physical activity practice opportunities, contributing to healthy lifestyles, but also showing evidence of benefits for physical, social, mental, and community health. In this issue, Rasmussen et al. [3] question how Nike sport advertisements can encourage individuals to engage in sports participation, with a focus on gender, and Pedersen et al. [4] have reviewed literature on how different social backgrounds have importance in sport participation.

In health promotion through sport, it is emphasized that sport is used as a means, a tool or a vehicle for other outcomes than sport, or in combination with sport outcomes. Here, sport-for-development, sport-for-peace, positive youth development, and sport management studies are examples. These studies seldom use the concept of health, but they focus on the determinants of health, such as social responsibility. In this issue, Sanchez et al. [5] explain residents’ perceptions of the corporate social responsibility of sports events, and Escamilla-Fajardo et al. [6] study entrepreneurial orientation as a way to manage sports clubs during times of change. Both studies are in Spanish.

Studies included in the health promotion in sport cluster use the setting of sport to reach target groups for specific health outcomes, often lifestyle habits, arranging specific activities, programs, or interventions. The initiatives can be on different levels, from the education of participants and parents, or coaches to more organizational initiatives, such as health sponsorship. In this issue, Chaugé et al. [7] investigate the prevalence of smoking in amateur rugby clubs in France, and the need for health promotion interventions to prevent this. Rato Barrio et al. [8] have mapped the literature on how to promote mental health in adult athletes through coaches.

In the area of health-promoting sports, the focus is both on sports and health promotion, where the whole systems are looked upon from health-promoting perspectives, considering organizational, cultural, social, and environmental determinants of health. In this issue, Van Hoye et al. [9] provide an illustration of health promotion initiatives in sports clubs in France, and Lane et al. [10] present a follow-up study of the Healthy club project of the Gaelic Athletic Association in Ireland. Carrad et al. [11] explain how the gymnastic federation in Australia changes sports clubs’ health promotion. In Donaldson et al. [12], the challenges of partnering to promote health through sport in Australia is covered, and in Ooms et al. [13], they explore whether health-promoting programs for inactive people in the Netherlands can lead to health-promoting sports. Finally, in the last two papers, the salutogenic health promotion theory is applied to explore young peoples’ perspectives on health-promoting sports in Sweden, first by Thedin Jakobsson et al. [14], and then by Geidne et al. [15], in combination with the settings-based approach to health promotion.

Still, there are of course remaining challenges regarding the association between health promotion and sport, for example the acknowledgement of these definitions and their stakes, as well as the recognition of health promotion and sport as a research field. Further challenges include a need to encompass a broad diversity of disciplines (e.g., sport management, medicine, and psychology), considering sport as a system, even though specific sports and contexts have specificities, and taking into account the broad diversity of health determinants, and the diversity of actions (from social inclusion to sustainable development, including injury prevention and more).

As a conclusion of the Special Issue, we would argue that the concept of health promotion is often used in a narrow sense, or as something other than what it is defined as. When studying the relationship between health promotion and sport without using definitions of health promotion and sport, researchers tend to miss important dimensions. There is furthermore a vast amount of research related to health promotion and sport, with a common vision to develop sport. However, they often originate from different disciplines and use different theories, methodologies, and definitions of sport and health. These different research branches almost never refer to each other, which hampers cumulative knowledge development. We would argue that the potential lies in embracing a considerable bank of knowledge on how sports can be developed in healthy ways. So, what if we started to look more into how these different branches can learn from each other?

## Data Availability

Not applicable.
